# A 180 nm Self-biased Bandgap Reference with High PSRR Enhancement

**DOI:** 10.1186/s11671-020-03333-w

**Published:** 2020-05-11

**Authors:** Yue Shi, Shilei Li, Jianwen Cao, Zekun Zhou, Weiwei Ling

**Affiliations:** 1 College of Communication Engineering (College of Microelectronics), Chengdu University of Information Technology, Chengdu, 610225 China; 2 State Key Laboratory of Electronic Thin Films and Integrated Devices, University of Electronic Science and Technology of China, Chengdu, 610054 Sichuan China

**Keywords:** Global self-biased loop, Local negative feedback loop, High power supply rejection ratio

## Abstract

In this paper, an improved self-biased bandgap reference (BGR) with high power supply rejection ratio (PSRR) is presented. An operational amplifier constructing feedback loop is multiplexed with the generation of positive temperature coefficient (TC) voltage for lower power consumption, where an offset voltage is adopted to achieve proportional to absolute temperature (PTAT) voltage. With the temperature-independent reference generation, two feedback loops are realized at the same time for PSRR enhancement, which form a local negative feedback loop (LNFL) and a global self-biased loop (GSBL). The proposed BGR is implemented in a 180 nm BCD technology, whose results show that the generated reference voltage is 2.506 V, and the TC is 25 ppm/°C in the temperature range of −55 to 125 °C. The line sensitivity (LS) is 0.08 ‰/V. Without any filter capacitor, the PSRR is 76 dB at low frequencies, over 46 dB up to 1 MHz.

## Introduction

Voltage reference is one of the core modules in electronic systems, which is widely used in medical electronics, power managements, wireless environmental sensors, and communication circuits. With the improvement of technology, the area of chip continues to shrink, and the anti-interference ability continues to increase, and the requirements for structural optimization and noise immunity of voltage reference are increasing dramatically, especially in nanoscale applications [1].

Conventional bandgap reference (BGR) circuits require additional circuit blocks to provide bias current for the entire circuit, which greatly increases the circuit area and power consumption. At the same time, the generated bias current is greatly affected by temperature, which affects the temperature coefficient (TC) of the reference voltage. Lots of high-order compensated techniques for improved TC have been reported, such as piecewise curvature compensation [2], exponential curvature compensation [3], leakage-based square root compensation (LSRC) [4], and so on. Another disadvantage of the conventional BGR circuit is that it is greatly affected by the external environment and the output voltage is unstable, which is the focus of this article.

Power supply rejection ratio (PSRR) is an important parameter to measure the noise immunity of a voltage reference. Conventional solutions to improve PSRR are at the cost of chip area and power consumption [5], such as additional amplifiers, long channel transistors, cascode structures [6], additional gain stage [7], and so on. Active attenuator and impedance adapting compensation were adopted in [8] to improve the PSRR at low and high frequencies, respectively. Yue et al. [9] used cascode current mirrors to enhance PSRR. Body bias and negative feedback techniques were utilized in [10] for high PSRR.

In order to overcome the above-mentioned issues, an improved self-biased BGR with high PSRR is proposed in this brief. Two feedback loops are realized at the same time for PSRR enhancement, which form a local negative feedback loop (LNFL) and a global self-biased loop (GSBL). Meanwhile, a self-bias current source (SBCS) for the whole BGR is achieved. At steady state, the proposed BGR is self-powered through the GSBL without additional bias current modules and chip area. The presented technique separates the supply voltage from the output reference voltage through a current amplifier embedded in GSBL, which can effectively improve the PSRR. In addition, in order to prevent the output voltage from instability, a LNFL is designed at the output voltage terminal to keep the output voltage stable. What is more, the temperature-stable reference voltage is generated with LNFL and GSBL in a multiplexing way. With these methods, a self-biased BGR with high PSRR enhancement is implemented with compacted structure and current consumption.

## Method

**Fig. 1:**
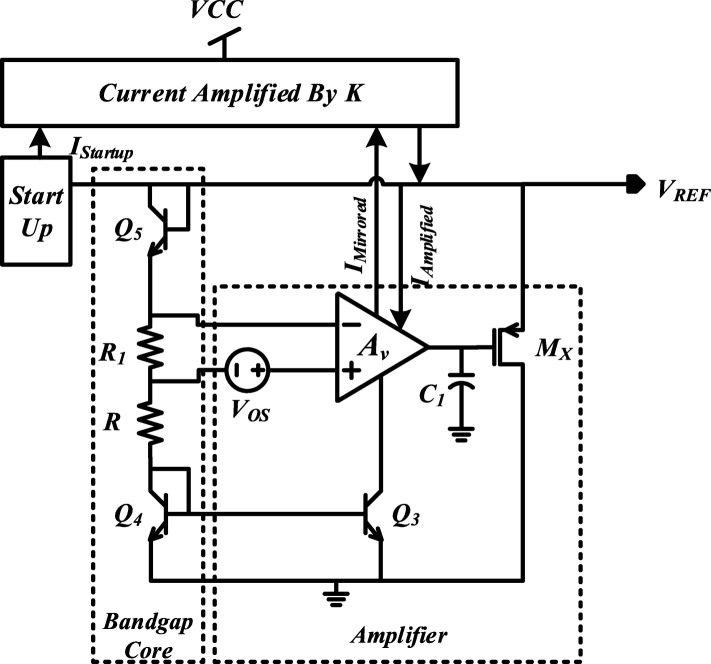
Equivalent architecture diagram of proposed voltage reference

**Fig. 2:**
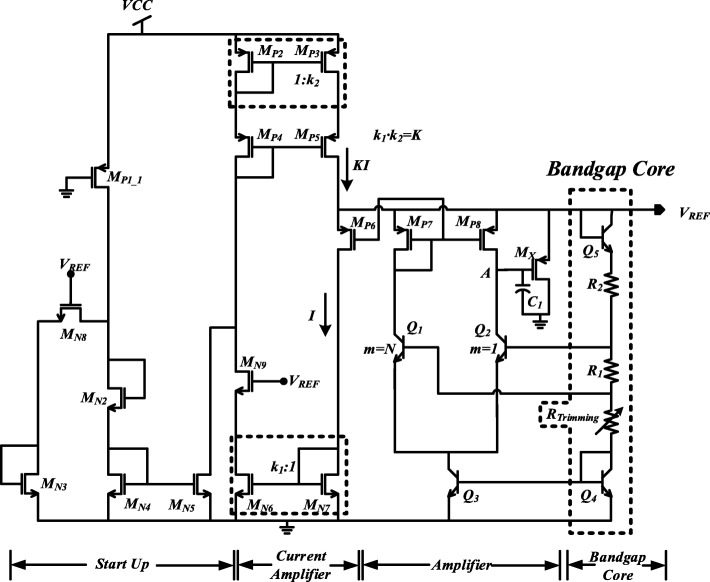
Schematic of proposed voltage reference

As shown in Fig. 1, the proposed BGR circuit consists of a start-up circuit, a current amplifier, an operational amplifier, and a bandgap reference core. The start-up circuit is used to get rid of the zero-degenerate point. The built-in offset voltage in the amplifier is set to be proportional to absolute temperature (PTAT) voltage, which can realize a PTAT current through resistor R1. With the positive TC of voltage across R1 and R2, the negative TC of *V*_BE(Q5)_ and *V*_BE(Q4)_ can be properly canceled to achieve a temperature-stable reference voltage at node *V*_REF_. At the same time, a LNFL formed with the help of an amplifier to improve the performance. Combined with the current amplifier on the top of Fig. 1, a GSBL is realized for further PSRR improvement. The detail implementation of the proposed BGR is shown in Fig. 2.

### Start-up Circuit

The start-up circuit is shown in the left part of Fig. 2. At the beginning of the start-up stage, output voltage *V*_REF_ is at low level, which keeps MN8 and MN9 off. The current through MP1_1 is used to generate a start-up current to MP5, where MP1_1 is as a large resistance with quite small aspect ratio. The voltage at *V*_REF_ will be gradually charged by the start-up current. When the voltage at *V*_REF_ exceeds the minimum operation voltage of the bandgap core part, the bias current for the amplifier will be generated. This will drive the BGR to the desired operation point. At the same time, transistors MN8 and MN9 will be gradually on, which switches the supply current of MP5 to the self-biased current generated in the bandgap core. After the startup is completed, the startup current is not turned off for *V*_REF_ re-adjustment in the case of reference voltage falling for some reasons [11].

### SBCS Generator

There are two SBCS loops in the proposed BGR, which are helpful for performance enhancement [1]. The first one is located at the tail current of the amplifier. The PTAT current through transistor Q4 is mirrored into Q3. However, the current through Q4 is determined by the voltage across resistor R1, which is clamped to the input offset voltage of the amplifier. Due to the same aspect ratios of MP7 and MP8, the input offset voltage of the amplifier can be expressed as

1 $$ {V}_{OS}={V}_T\ln N $$

where *N* is the area ratio of Q1 and Q2, and *V*_*T*_ is the thermal voltage. Therefore, the current in amplifier and bandgap core parts is PTAT current, which can be given by

2 $$ {I}_{R1}={V}_T\ln N/{R}_1 $$

The current of bandgap reference core is mirrored into the amplifier as tail current, forming the first self-biased loop.

The second SBCS loop is made up with the current amplifier. The PATA current shown in equation (2) is mirrored into the current amplifier by the current mirror of MP7 and MP6. Then the current, *I*, is amplified by *K* as the current source to node VREF, which can be described as

3 $$ K={k}_1{k}_2 $$

where *k*_1_ = *S*_*MN*6_/*S*_*MN*7_, *k*_2_ = *S*_*MP3*_/*S*_*MP2*_, *S*_*i*_ is the aspect ratio of transistor *i*. Therefore, the current, *KI*, is re-injected into the amplifier and bandgap core parts, which constructs the second self-bias loop.

In order to guarantee the proper operation with low power consumption, the current, *KI*, should be slightly larger than the minimum current requirement of amplifier and bandgap core. In the proposed design, the currents through MP6, MP7, and MP8 are set at the same level, *I*. The current through bandgap core is 2*I*. Therefore, the relationship, 6 ≥ *K* > 5, should be satisfied [12–14].

### *V*_*REF*_ Generator Circuit

The *V*_*REF*_ generator circuit is shown in the right part of Fig. 2, which consists of an amplifier and bandgap core. As shown in equation (2), the PTAT offset voltage of the amplifier is multiplexed by the SBCS loops [15]. This makes the current through R1, R2, and R_Trimming_ is PTAT current, which is used as temperature compensation of the negative TC of Q4 and Q5. The generated reference voltage, *V*_REF_, can be expressed as

4 $$ {V}_{REF}=2{V}_{BE}+\left(1+\frac{R_2+{R}_{Trim\min g}}{R_1}\right){V}_T\ln N $$

With the ratio adjustment of (*R*_2_ + *R*_*Trim* min *g*_)/*R*_1_, a temperature-compensated reference voltage can be realized with low-temperature drift.

### Feedback

A LNFL is established in the amplifier and bandgap core, which is formed by two small LNFLs. The first one, loop1, is from the input of the amplifier to *V*_REF_, and feedback to the input of the amplifier. The other one, loop2, is from *V*_REF_ through Bandgap core to current tail of amplifier, and feedback to *V*_REF_. For loop1, there are positive feedback and negative feedback double local loops with the input of the amplifier. The positive feedback loop is composed of Q5, R2, R1, Q1, MP8, and MX. The negative feedback loop consists of Q5, R2, Q2, and MX. The gain of the positive and negative feedback loop is derived as

5 $$ {A}_{V, PF}=\frac{R_{Trim\min g}}{R_1+{R}_{Trim\min g}+{R}_2}{g}_{m,Q1}{r}_{o, MP8} $$

6 $$ {A}_{V, NF}=\frac{R_1+{R}_{Trim\min g}}{R_1+{R}_{Trim\min g}+{R}_2}{g}_{m,Q2}{r}_{o, MP8} $$

where *g*_*m*, *Q*1_ is the transconductance of transistor Q1, *r*_*o*, *MP*8_ is the output resistance of transistor MP8, and the *g*_*m*_ of Q1 and Q2 is approximately equal. Since the effect of the negative feedback loop is stronger than that of the positive feedback loop, the loop1 behaves as a feedback loop, whose loop characteristic can be expressed as

7 $$ {T}_{\mathrm{loop}1}\approx \frac{R_1}{R_1+{R}_{Trim\min g}+{R}_2}{g}_{m,Q1}{r}_{o, MP8} $$

8 $$ {p}_0\approx \frac{1}{r_{o, MP8}{C}_1} $$

where *p*_0_ is the dominant pole. With regard to loop2, the performance can be given by

9 $$ {T}_{\mathrm{loop}2}\approx \frac{1/{g}_{m, MP8}}{R_1+{R}_{Trim\min g}+{R}_2} $$

10 $$ {p}_1\approx \frac{g_{m, MP8}}{C_1} $$

where *g*_*m*, *MP*8_ is the transconductance of transistor MP8, and *p*_1_ is the dominant pole. As a result, the total loop gain of LNFL is

11 $$ {T}_{\mathrm{LNFL}}\approx \frac{R_1{g}_{m,Q1}{r}_{o, MP8}+1/{g}_{m, MP8}}{R_1+{R}_{Trim\min g}+{R}_2}\frac{1+s/{z}_0}{\left(1+s/{p}_0\right)\left(1+s/{p}_1\right)} $$

Taken equation (2) into consideration, equation (11) can be rewritten as,

12 $$ {T}_{\mathrm{LNFL}}\approx \frac{r_{o, MP8}\ln N+1/{g}_{m, MP8}}{R_1+{R}_{Trim\min g}+{R}_2}\frac{1+s/{z}_0}{\left(1+s/{p}_0\right)\left(1+s/{p}_1\right)} $$

where *z*_0_ ≈ *g*_*m*, *MP*8_/[*C*_1_(1 + 1/ ln *N*)]. Since *N* = 8 in the proposed design, it makes the zero, *z*_0_, proximately equal to twice the pole, *p*_1_, which can extend the loop bandwidth of LNFL by twice.

A GSBL is formed by the current amplifier, bandgap core, and amplifier, which can provide bias current for the whole circuit in a self-biased method with enhanced PSRR performance. The loop gain of GSBL can be given by

13 $$ {T}_{\mathrm{GSBL}}\approx \frac{K\left(1/3{g}_{m, MP8}\Big\Vert 1/{g}_{m, MX}\right)}{R_1+{R}_{Trim\min g}+{R}_2} $$

where *g*_*m*, *MX*_ is the transconductance of transistor *M*_X_. The main effect of transistor *M*_X_ is to lower the equivalent impedance at *V*_REF_ with the convenience of loop compensation. *T*_GSBL_ is set to be smaller than one in the proposed design, which can avoid oscillation.

With the help of LNFL and GSBL, the stability of generated reference voltage, *V*_REF_, can be greatly improved.

### PSRR of Proposed Voltage Reference

**Fig. 3:**
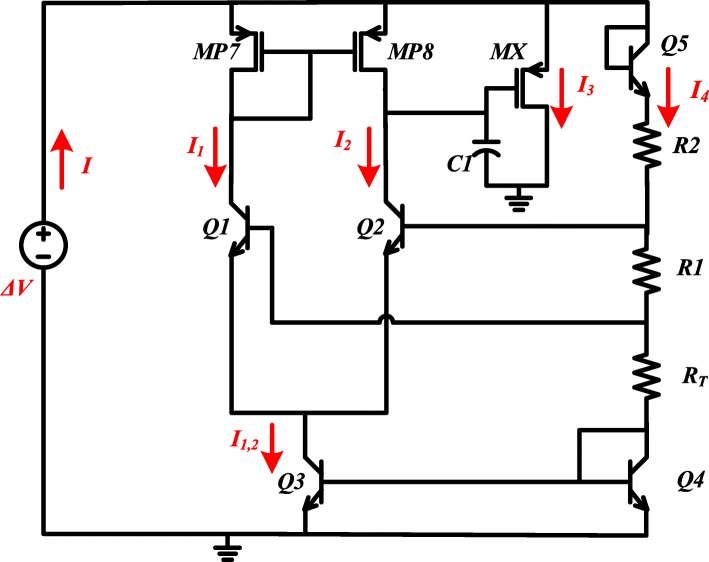
R_eq_ calculation diagram

In order to simplify the PSRR calculation of the proposed circuit, the equivalent resistance of the part powered by the reference voltage, *V*_*REF*_, is firstly calculated. The calculation diagram of this part is shown in Fig. 3 [16].

**Fig. 4:**
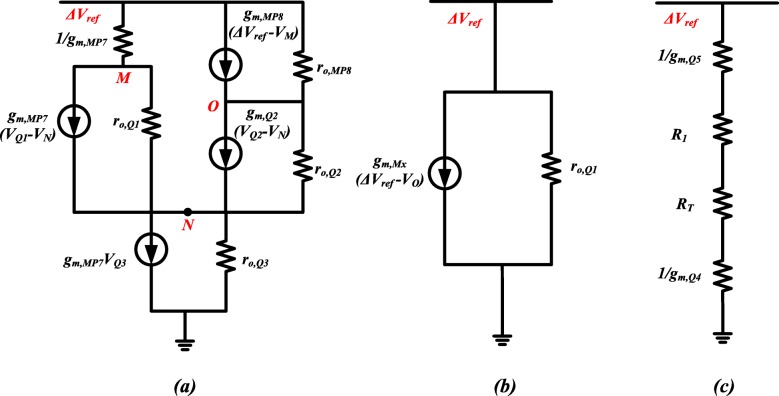
Small-signal model for R_eq_. **a** R_eq1,2_ calculation diagram. **b** R_eq3_ calculation diagram. **c** R_eq4_ calculation diagram

Figure 4a shows a small-signal model for the equivalent resistance calculation of circuit branches 1, 2, where currents *I*_1_ and *I*_2_ flow in Fig. 3, respectively. Then, the equivalent resistance, *R*_*eq*1,2_, can be expressed as

14 $$ {R}_{eq1,2}\approx \frac{3{R}_{eq,4}{r}_{o,Q1}}{3{g}_{m,Q1}{r}_{o,Q1}\left({R}_T+{R}_1+{r}_{o,Q3}\right)+{g}_{m,Q1}{R}_1{r}_{o,Q1}+3{R}_{eq,4}} $$

where *g*_*m*,*Q*1_ and *r*_*o*,*Q*1_ are transconductance and output resistance of Q1, respectively; *R*_*eq*4_ is equivalent resistance of branch with *I*_4_. Since the gate voltage of MP6 shown in Fig. 2 is determined by the drain voltage of MP7, the power supply noise attenuation (PSNA) at node M should be also calculated, which can be given by

15 $$ {V}_M=\Delta {V}_{ref}+\frac{g_{m,Q1}{R}_1{r}_{o,Q2}}{2{g}_{m, MP7}\left({r}_{o,Q2}+{r}_{o, MP8}\right){R}_{eq4}}\Delta {V}_{ref}\approx \Delta {V}_{ref} $$

where *r*_*o*,*MP*8_ and *r*_*o*,*Q*2_ are output resistance of MP8 and Q2, respectively; *g*_*m*,*MP*7_ is transconductance of MP7. As claimed in equation (15), the supply noise has little influence on the source-gate voltage of MP6. This makes MP6 act as a high impedance, *r*_*o*,*MP*6_, which separates the noise impacts from the amplifier and bandgap core parts.

The equivalent resistance of branch with *I*_3_ in Fig. 3 can be derived by Fig. 4b, which can be expressed as

16 $$ {R}_{eq3}\approx \frac{6{R}_{eq,4}}{g_{m, mx}\left[3{g}_{m,Q1}{r}_{o,Q1}\left({R}_T+{R}_1+{r}_{o,Q3}\right)+3{R}_{eq,4}+{g}_{m,Q1}{r}_{o,Q1}{R}_1\right]} $$

where *g*_*m*,*Mx*_ is the transconductance of Mx. The small-signal model of equivalent resistance of branch with *I*_4_ in Fig. 3 is shown in Fig. 4c, which is,

17 $$ {R}_{eq4}\approx 1/{g}_{m,Q5}+{R}_1+{R}_T+1/{g}_{m,Q4}+{R}_2 $$

Therefore, the small-signal equivalent resistance of amplifier and bandgap core parts in Fig. 3 is

18 $$ {R}_{eq}={R}_{eq1,2}\left\Vert {R}_{eq3}\right\Vert {R}_{eq4} $$

**Fig. 5:**
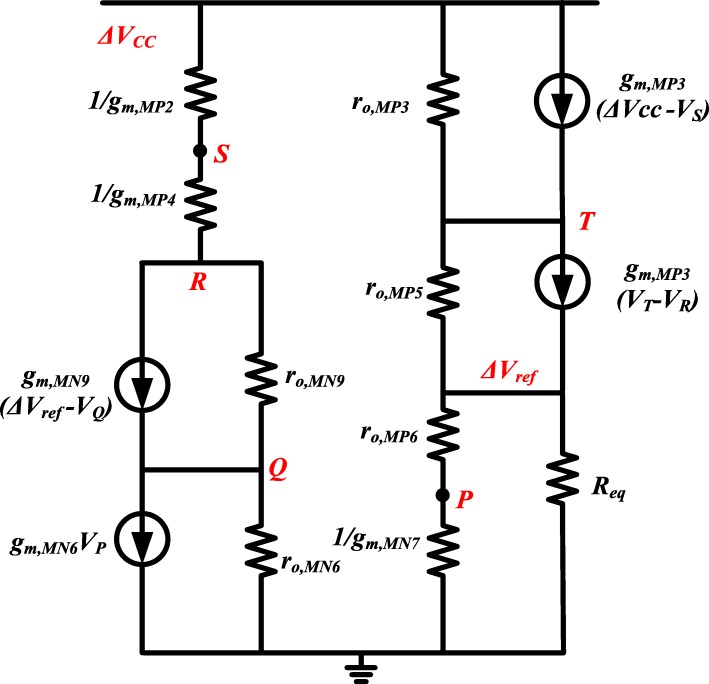
Small-signal model for PSRR

Therefore, the total PSRR of the proposed voltage reference can be illustrated in Fig. 5. The PSRR can be given by

19 $$ \frac{\Delta {V}_{ref}}{\Delta {V}_{CC}}\approx \frac{6{R}_{eq,4}}{g_{m, mx}{g}_{m, mp3}{r}_{o, mp3}{r}_{o, mp6}\left[3{g}_{m,Q1}{r}_{o,Q1}\left({R}_T+{R}_1+{r}_{o,Q3}\right)+3{R}_{eq,4}+{g}_{m,Q1}{r}_{o,Q1}{R}_1\right]} $$

Since *g*_*m*_*r*_*o*_ >  > 1 is generally valid, the influence of power supply noise on the generated reference voltage is greatly suppressed.

## Results and Discussion

**Fig. 6:**
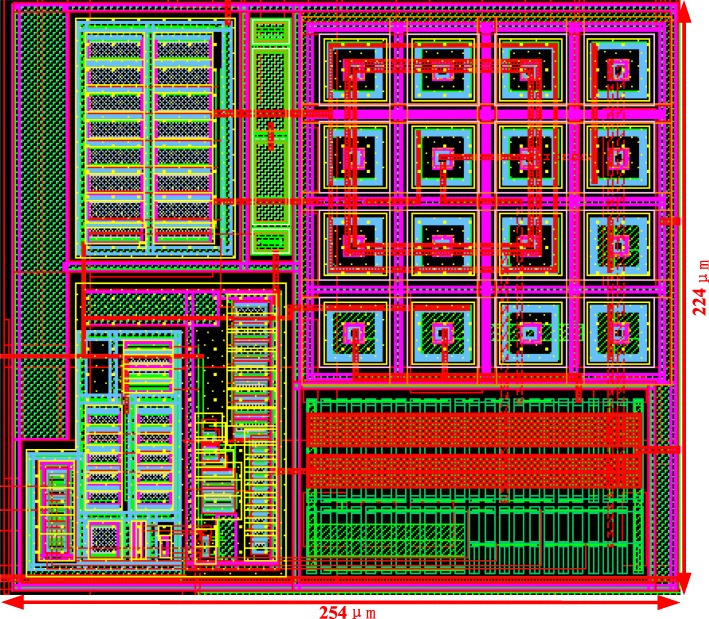
Layout of proposed circuit

The voltage reference is implemented in a 180 nm BCD process, whose layout is shown in Fig. 6, occupying a 0.05690 mm^2^ active area.

**Fig. 7:**
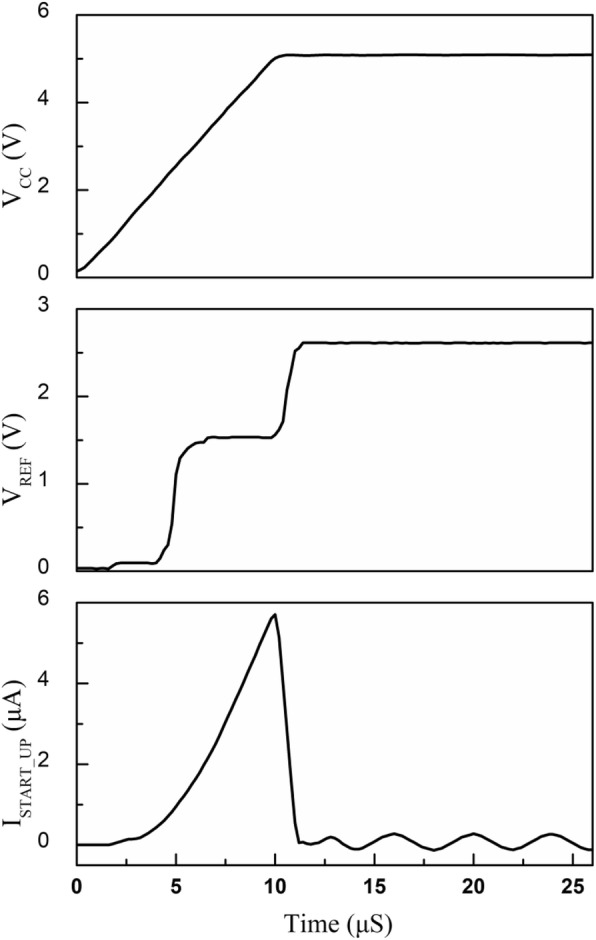
Start-up transient characteristic of proposed voltage reference

The simulated start-up waveforms are shown in Fig. 7, which illustrates the transient procedure with the power-supply-voltage establishment. When the supply voltage is small, the entire reference circuit is not fully operated, which means the startup branch current is very small and the reference voltage is maintained at zero. With the rising of power supply voltage, the generated reference voltage is firstly stable at approximately 2*V*_BE_ due to the abnormal operation of the amplifier part in Fig. 2. When the supply voltage increases above the minimum required supply voltage of proposed BGR, the core operational amplifier starts to work, and the reference voltage is quickly stabilized at the desired value. Besides, the start-up current drops about to zero with a desired reference voltage, while the proposed SBCS taking the place of current supply with the GSBL. The power consumption of start-up circuit accounts for a small part of that of the chip.

**Fig. 8:**
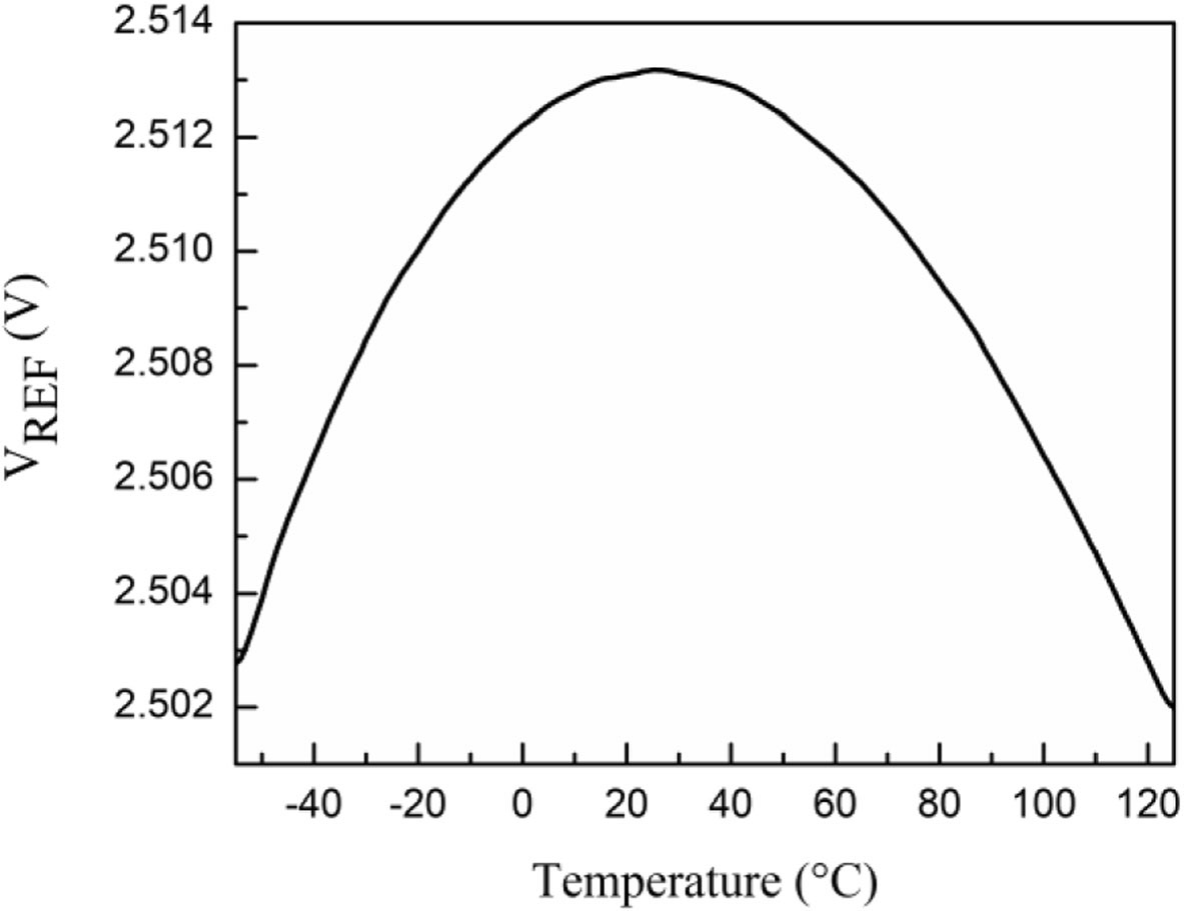
Temperature dependence of generated reference voltage

The temperature characteristics of the generated reference voltage, *V*_*REF*_, are shown in Fig. 8. The voltage variation of *V*_*REF*_ in the range of −55 °C ~ 125 °C is 11.3 mV, where a TC of 25 ppm/°C is achieved.

**Fig. 9:**
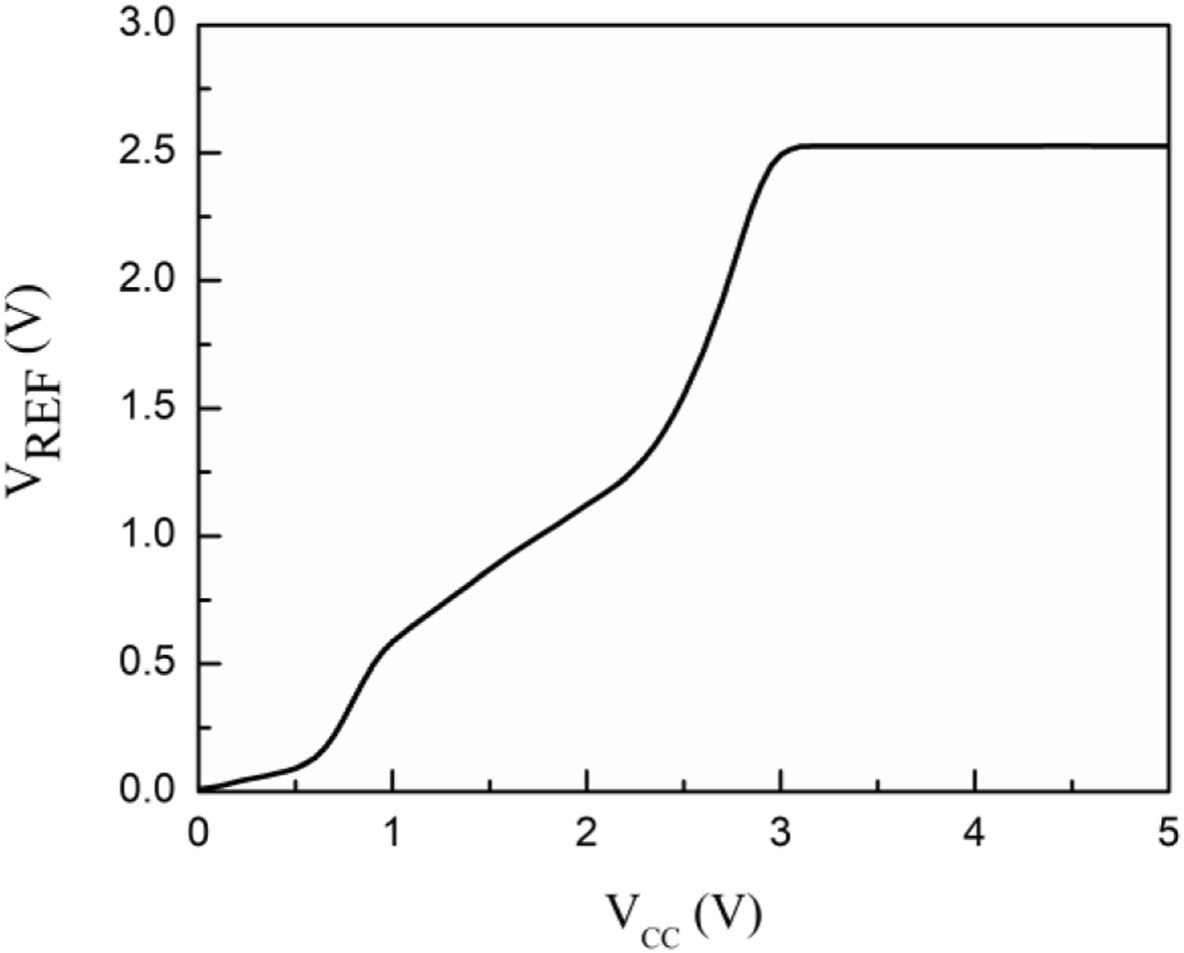
Supply dependence of generated reference voltage

Figure 9 demonstrates the line sensitivity (LS) of the reference output voltage. The proposed BGR can be successfully established over 3 V supply voltage, and *V*_*REF*_ variation is 0.2 mV within 3 -5 V supply voltage. This means a good LS of 0.08‰/V is realized.

**Fig. 10:**
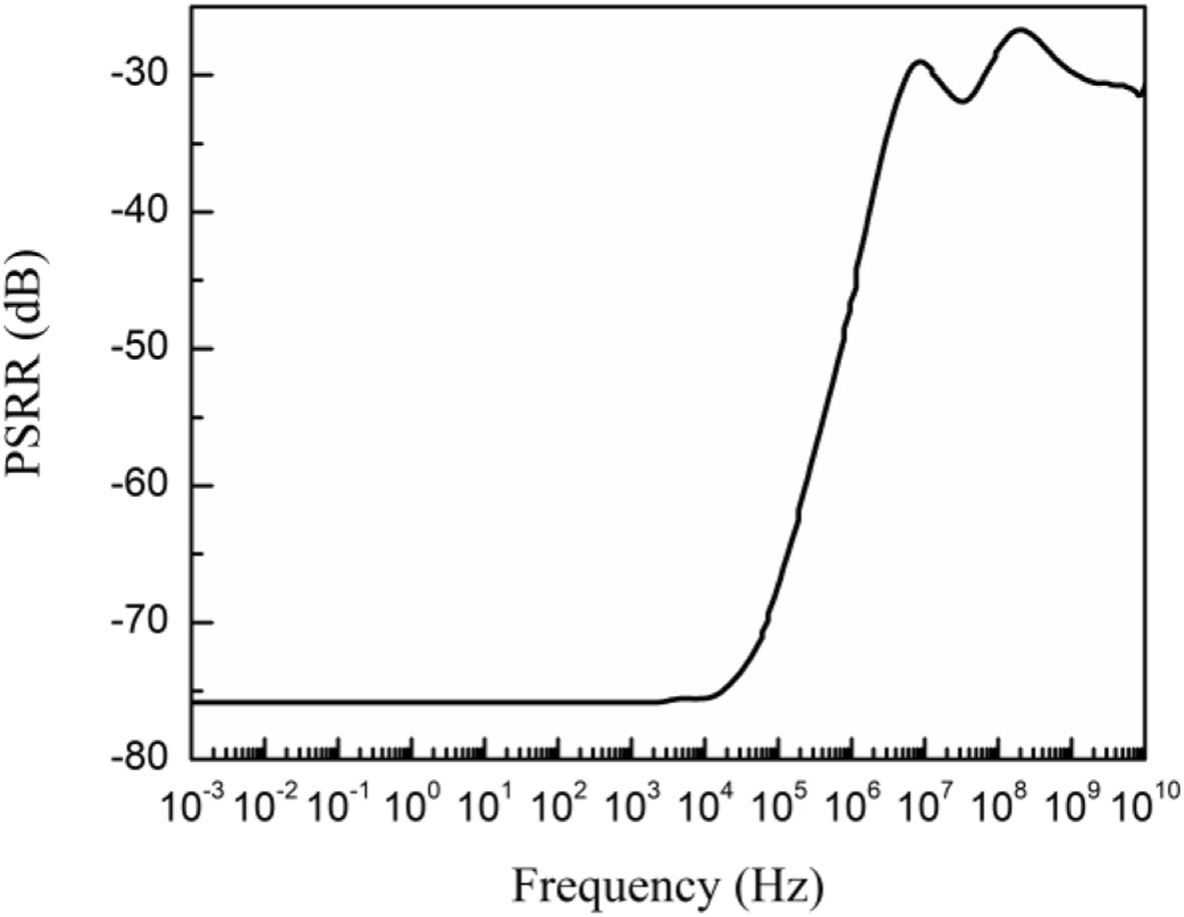
PSRR characteristic of proposed voltage reference

The improved PSRR performance is illustrated in Fig. 10, which has a PSRR of 76 dB agreeing with theoretical results in equation (19) at low frequencies and above 46 dB up to 1 MHz.

Conventional binary trimming method is suitable for the proposed BGR, which adopts an 8-bit trimming for *R*_Trimming_. This can realize a 9 mV/LSB trimming step. Table 1 shows the performance of trimmed voltage reference with 3 -5 V supply voltage and −55 to 125 °C temperature range under difference process corners, which include typical, slow, and fast cases. As shown in Table 1, the temperature drift is within 0.6%, the LS is below 0.12‰/V, and PSRR is above 71 dB@10 Hz.

**Table 1:** Performance of reference voltage with process variations

Parameter results	*V*_REF_ (*V*)	TC (ppm/°C)	LS (‰/V)	PSRR@10 Hz (dB)
Minimum value	2.484	24	0.02	−71
Typical value	2.506	25	0.08	−76
Maximum value	2.522	30	0.12	−78

Table 2 gives the characteristic summary of the proposed voltage reference and the comparison with some previously reported voltage references. Since the proposed voltage reference is aiming at high supply stability, no high-order temperature compensation is utilized in this paper. Therefore, the TC of [11–13], which mainly focus on temperature or power optimization methods, is smaller than that of the proposed voltage reference. The TC of the proposed voltage reference can be further optimized with literature reported curvature-compensation methods as needed. With the proposed compacted structure, LNFL and GSBL are realized with a temperature-independent reference voltage at the same time, which has the best PSRR and LS performance in Table 2.

**Table 2:** Performance summary and comparison

	This work	[17]	[18]	[19]	[20]
Process (nm)	180	350	180	180	180
Minimum supply voltage (V)	3	2	1.4	0.9	0.8
Power (W)	45 μ	66 μ	34p	85n	79n
Temperature range (°C)	−55-125	−40-125	0-100	−40-125	10-100
V_REF_ (V)	2.506	1.141	1.250	0.412	0.328
TC (ppm/°C)	25	1.01	23	33.7	33.8
PSRR@10 Hz (dB)	−76	−61	−42.2	−61	−55
LS (‰/V)	0.08	2	2.5	0.6	2.1

## Conclusion

A compacted self-biased BGR with high PSRR is presented in this paper. The PTAT voltage is implemented by an operational amplifier with asymmetrical input offset voltage, and the negative temperature voltage is superimposed to generate a reference output voltage. At the same time, two feedback loops, LNFL and GSBL, are realized with the same parts for temperature stability, which reduces the structural complexity. This leads to self-sufficiency of supply current and power supply sensitivity improvement with high PSRR.

## Data Availability

All data generated or analyzed during this study are included in this published article.

## Abbreviations

BGR: Bandgap reference
PSRR: Power supply rejection ratio
TC: Temperature coefficient
PTAT: Proportional to absolute temperature
LNFL: Local negative feedback loop
